# Effectiveness of mental practice for limb dysfunction in patients who have experienced a stroke: a systematic review and meta-analysis

**DOI:** 10.12968/bjnn.2023.19.Sup2.S23

**Published:** 2023-05-03

**Authors:** Oliver Hamer, James Edward Hill, Mariya Zafar, Natalie Morgan

**Affiliations:** 1 University of Central Lancashire, Preston, UK; 2 East Lancashire Hospital Trust

**Keywords:** Mental practise, Intervention, Stroke, Imagery, limb dysfunction, Systematic review

## Introduction

Stroke is the second largest cause of death in adults and the principal cause of long‐term severe adult disability worldwide (GBDS 2021). Globally, there are approximately 5.5 million deaths attributed to stroke each year (GBDS 2021). As a consequence of stroke, a large proportion of individuals suffer impairments such as limb weakness, urinary incontinence, impaired consciousness, dysphagia, and impaired cognition (Lawrence et al. 2001; Stockley et al. 2021). One of the more frequent impairments is upper limb weakness, whereby approximately 70% of patients present following a stroke (Lawrence et al. 2001; Nakayama et al. 1994). Upper limb dysfunction effects mental health and quality of life (Lieshout et al. 2020). It has shown to limit the level of independence and engagement in hobbies, employment, and daily activities (Lieshout et al. 2020). Improving arm function is a core element of rehabilitation for patients with upper limb dysfunction following stroke (Pollock et al. 2014a). Many interventions have been developed to achieve this which typically involve different exercises or training (e.g., repetitive task practice, constraint-induced movement therapy), cognitive practise (e.g., mental practise, mirror therapy) or pharmacological medication (Pollock et al. 2014a).

One intervention for treatment of upper limb impairment post-stroke, is mental practice (MP) (Page et al. 2007). Mental practice is an intervention (delivered primarily by a clinical psychologist) where participants are guided to cognitively rehearse, but not physically perform movements of the upper limb (Page et al. 2007). The intervention focuses on the cognition of how to complete a functional task (Page et al. 2007). An example of this would be the mental practise of gripping an item, were patients repeatably visualise the action. Although mental practise is included within stroke guidelines, it is not a commonly used intervention due to the lack of guidance around how and with whom the intervention should be used (Malouin et al. 2009; Pollock et al. 2014b). A 2020 Cochrane systematic review in 2020 established mental practise as an effective intervention, however, implementation was hindered by a lack of reporting around dose and whom the intervention may benefit most (Barclay et al. 2020). A recent systematic review by Stockley et al, 2021 has examined the effectiveness of MP on outcomes of activity limitations, describing when and in whom MP might have the most benefit to upper limb activity outcomes after stroke (Stockley et al. 2021).

### Aim of commentary

This commentary aims to critically appraise the methods used within the review by Stockley et al, 2021, and expand upon the findings in the context of clinical practice (Stockley et al. 2021).

## Methods

This PROSPERO registered systematic review carried out a robust multi-database literature search from November 2009 to May 2020. Previous studies prior to 2009 were identified from a previous Cochrane review by Barclay-Goddard et al, (Barclay-Goddard et al. 2011). Only studies published in English were included. Only random controlled trials which included adults with a confirmed diagnosis of stroke, - had a sensorimotor upper-limb involvement resulting from stroke -and received MP were included in the review. A robust screening, data extraction and quality assessment was undertaken by two independent reviewers. Any uncertainty about suitability for inclusion was discussed between two reviewers until an agreement was reached. PEDro criteria scores were utilized to assess the quality of the included studies. Published assessments on the PEDro website were used to indicate the quality of the included studies where possible. Where appropriate, a random-effects meta-analysis was undertaken using standardised means difference and means difference. Funnel plots of effect estimates from studies against a measure of precision were used to assess risk of bias. Subgroup analyses were undertaken for time after stroke onset, severity of upper limb and dose.

## Results

After duplicate removal 1,239 citations were identified of which after full paper screening 15 studies were included and 12 were appropriate to be meta-analysed. These 15 studies included 465 people (n=282 & women, n=183) with a mean age of 59.2 years old.

### Overall effectiveness of MP

When meta-analysed using a random effects model, there was a clinical and statistically significant moderate effect for improving activity limitations of the upper limb across all time periods compared to conventional therapy, usual care, or placebo intervention (control group) [7 moderate quality & 5 high quality RCTs].

### Time of outcome measurement

When meta-analysed, MP demonstrated a clinical and statistically significant large effect on measures of activity limitations in the early subacute (7 days to 3 months after stroke) period compared to the control group [2 moderate quality & 1 high quality RCTs]. Following this period, MP was less effective with a clinically and statistically significant moderate effect being observed during the chronic period (≥six months) [4 moderate quality & 3 high quality RCTs]. However, there was no evidence of effect for the late subacute period (3 to 6 months) [2 high quality RCTs].

### Upper limb limitations

Greatest effect of MP was observed for those who had the greatest degree of upper limb dysfunction. With patients with a baseline Action Research Arm Test (ARAT) score between 0-20 scoring the highest comparative score (ARAT scores: weighted mean difference [WMD], 7.33; 95% CI, 0.94 to 13.72, 1 moderate quality & 2 high quality RCTs) followed by patients with an ARAT scores, 21-40 (ARAT scores: WMD, 5.13; 95% CI, 2.88 to 7.39, 3 moderate quality & 1 high quality RCTs). There was no evidence that MP was effective for those with patients with an ARAT scores 21-40 (ARAT scores: WMD, 2.50; 95% CI, -4.38 to 9.38). However, this is based upon one moderate quality RCT.

### Dose response

There was no evidence of a dose response. ≤ 6.6 minutes a day of MP produced a statistically significant large effect [1 moderate quality & 1 high quality RCTs], 6.7 to 32 producing a statistically significant moderate effect [3 moderate quality & 2 high quality RCTs] and ≥ 32.1 minutes a day of MP producing a statistically significant moderate effect [2 moderate quality & 3 high quality RCTs]. However, it is important to note that at all three doses there were a wide confidence interval and large overlap.

### Commentary

Using the AMSTAR-2 critical appraisal tool for systematic reviews, 14 criteria out of 16 were judged to be satisfactory (Table 1) (Shea et al. 2017). The review did not report funding sources for each included study and did not conduct a sensitivity analysis to assess the potential impact of RoB (individual studies) on the results of the meta-analysis. But it is unlikely that these particular criteria would notably alter the findings of this review. Thus, this systematic review provides an accurate and comprehensive summary of the evidence from existing studies available in the literature.

In terms of implementation, mental practise is likely to be most effective in the early subacute (7 days to 3 months after stroke) period on outcomes of activity limitations, less effective during the chronic period (≥six months after stroke), and may show no evidence of effect during the late subacute period (3 to 6 months after stroke). Therefore, when undertaking MP, it is important for clinicians to utilise this technique as early as possible (following a patient stroke) to gain the maximum beneficial effects.

When triaging patients who may benefit from MP, those who have the greatest degree of upper limb dysfunction may find the greatest benefit from this technique. As evident in the included studies, the application of Action Research Arm Test (ARAT) tool may be appropriate to identify these patients, as this tool has been previously demonstrated to be reliable and valid (Pike et al. 2018). Patients with a score between 0-20 should be seen as high priority for MP. Furthermore, clinicians should be conscious to select patients with normal or only mild cognitive dysfunction, given that effectiveness of MP for patients with reduced cognition has not yet been established (Stockley et al. 2021).

Regarding the dose of treatment, it is difficult to say the exact dose requirement due to the wide confidence intervals for MP at various time periods. Therefore, a pragmatic approach should be taken based upon the capacity of those who would be delivering the treatment. That said, the evidence suggests that even at low doses of less than six minutes a day, MP has been shown to demonstrate a clinically significant effect.

A limitation of the current synthesis was the lack of specific detail regarding the content and delivery of the MP intervention. As a consequence, it is challenging to generate policy and evaluate compliance or fidelity of MP interventions. Future research could establish a standardisation for the mode, dose, and delivery of how MP is implemented in clinical interventions. Future trials should also seek to compare the effectiveness of low to high doses of MP in studies of upper limb rehabilitation. Furthermore, research should consider broadening inclusion criteria to incorporate patients with cognitive deficits after stroke, to determine if they may benefit from MP.

### CPD reflective questions

- What are the key limitations of the systematic review discussed in this commentary and what needs to be considered when applying the evidence to practice?
- What factors are important when using MP?
- Are there any clinicians in your clinical area which currently practice MP which you could shadow and learn from?

## Figures and Tables

**Table 1:** Critical appraisal using the AMSTAR-2 tool for assessing systematic reviews

AMSTAR 2 items	Responses
1. Did the research questions and inclusion criteria for the review include the components of PICO?	Yes − the study outlined the participants, intervention, comparator, and outcomes.
2. Did the report of the review contain an explicit statement that the review methods were established prior to the conduct of the review and did the report justify any significant deviations from the protocol? Yes	Yes − the protocol was registered on Prospero (Ref: RD42019126044)
3. Did the review authors explain their selection of the study designs for inclusion in the review?	Yes - studies were included if they were a parallel group randomized controlled trial.
4. Did the review authors use a comprehensive literature search strategy?	Yes - Electronic searches of the following databases were completed: Cochrane Central Register of Controlled Trials, MEDLINE, EMBASE, CINAHL, PsycINFO, Scopus, Web of Science, the Physiotherapy Evidence Database (PEDro), the specialist rehabilitation research databases (CIRRIE), and REHABDATA.
5. Did the review authors perform the study selection in duplicate?	Yes − studies selection was independently conducted by 2 reviewers
6. Did the review authors perform data extraction in duplicate?	Yes - Data extraction was conducted by one reviewer and checked by a second reviewer
7. Did the review authors provide a list of excluded studies and justify the exclusions?	Yes - the author provided reasons for exclusion but did not list the studies.
8. Did the review authors describe the included studies in adequate details?	Yes − A characteristics of included studies was available in the publication
9. Did the review authors use a satisfactory technique for assessing the risk of bias in the individual studies that were included in the review?	Yes - Two reviewers independently assessed all included studies
10. Did the review authors report on the sources of funding for the studies included in the review?	No − the authors did not report funding sources for each study in the publication
11. If meta-analysis was performed did the review authors use appropriate methods for statistical combination of results?	Yes - Meta-analysis was conducted on the different continuous measures of upper limb activity, presenting results as point estimates and 95% confidence intervals (CI)
12. If meta-analysis was performed did the review authors assess the potential impact of RoB in individual studies on the results of the meta-analysis or other evidence synthesis?	No - the study did not conduct a sensitivity analysis to assess the potential impact of RoB in individual studies on the results of the meta-analysis.
13. Did the review authors account for RoB in individual studies when interpreting/discussing the results of the review?	Yes − the authors discussed the results in relation to the quality of evidence (i.e., moderate to high quality trials)
14. Did the review authors provide a satisfactory explanation for and discussion of, any heterogeneity observed in the results of the review?	Yes − the authors stated that heterogeneity was low in the meta-analyses.
15. If they performed quantitative synthesis did the review authors carry out an adequate investigation of publication bias(small study bias) and discuss its likely impact on the results of the review?	Yes − a funnel plots suggested that the findings of this systematic review may be skewed by publication bias.
16. Did the review authors report any potential sources of conflict of interest, including any funding they received for conducting the review?	Yes - The authors reported no competing interests

## Key Points

- MP has a moderate effect on improving activity limitations of the upper limb after stroke.
- Those with the greatest upper limb dysfunction gain the greatest benefit from mental practice.
- Where possible, mental practice should be utilised as early as possible after stroke.
- Future research is required on the standardisation of the mental practice approach, dose, and delivery of how it is implemented in clinical interventions.
